# Targeting RNA by Small Molecules: Comparative Structural and Thermodynamic Aspects of Aristololactam-β-D-glucoside and Daunomycin Binding to tRNA^phe^

**DOI:** 10.1371/journal.pone.0023186

**Published:** 2011-08-16

**Authors:** Abhi Das, Kakali Bhadra, Gopinatha Suresh Kumar

**Affiliations:** Biophysical Chemistry Laboratory, Indian Institute of Chemical Biology, Council of Scientific and Industrial Research, Kolkata, West Bengal, India; University of South Florida College of Medicine, United States of America

## Abstract

**Background:**

Interaction of aristololactam-β-D-glucoside and daunomycin with tRNA^phe^ was investigated using various biophysical techniques.

**Methodology/Principal Findings:**

Absorption and fluorescence studies revealed that both the compounds bind tRNA^phe^ non-cooperatively. The binding of daunomycin was about one order of magnitude higher than that of aristololactam-β-D-glucoside. Stronger binding of the former was also inferred from fluorescence quenching data, quantum efficiency values and circular dichroic results. Results from isothermal titration calorimetry experiments suggested that the binding of both compounds was predominantly entropy driven with a smaller but favorable enthalpy term that increased with temperature. A large favorable electrostatic contribution to the binding of daunomycin to tRNA^phe^ was revealed from salt dependence data and the dissection of the free energy values. The electrostatic component to the free energy change for aristololactam-β-D-glucoside-tRNA^phe^ interaction was smaller than that of daunomycin. This was also inferred from the slope of log K versus [Na^+^] plots. Both compounds enhanced the thermal stability of tRNA^phe^. The small heat capacity changes of -47 and -99 cal/mol K, respectively, observed for aristololactam-β-D-glucoside and daunomycin, and the observed enthalpy-entropy compensation phenomenon confirmed the involvement of multiple weak noncovalent interactions. Molecular aspects of the interaction have been revealed.

**Conclusions/Significance:**

This study presents the structural and eneregetic aspects of the binding of aristololactam-β-D-glucoside and daunomycin to tRNA^phe^.

## Introduction

The biological macromolecule RNA has attracted recent attention for its key role in various steps of gene expression. The recent knowledge of the essential role of cellular RNAs in several critical life processes and in the progression of many diseases, particularly viral infections like HIV, AIDS, and hepatitis C, has led to a growing interest in the molecule as a potential target for therapeutic intervention [1]–[3]. This has been augmented by the recent discovery of a number of micro RNAs and unraveling of their various functions [4], [5]. Consequently, a paradigm shift of interest from DNA binding molecules to RNA binding molecules has occurred recently, focusing intense research for the design and development of new RNA targeted therapeutic agents [6]–[9]. A rational design of RNA targeted small molecule therapeutics essentially requires both detailed knowledge of the structural details of RNAs and the molecular nature of the interaction phenomena. RNA binding studies have been severely hampered due to the lack of high-resolution conformational information and the complex RNA structures. However, recent advancements in structural aspects of RNAs through X-ray, NMR and computational studies have enabled detailed studies on the elucidation of the mode and mechanism of interaction of many new RNA binding molecules [10], [11]. For example, the aminoglycosides are a group of extensively studied RNA binding drugs that interact with the functional sites on 16 s of rRNA [12]–[14]. Although aminoglycoside antibiotics target key RNA molecules of bacteria and viruses, their therapeutic use is severely limited due to high toxicity [15]. We have been studying the interaction of a group of natural alkaloids with RNA targets like tRNA and poly(A) [16]–[19]. It was revealed that natural isoquinoline alkaloids constitute a group of molecules that may be useful in selectively targeting RNA through various means [20]–[23] Needless to say, RNA molecules have highly versatile structures that can fold into a multitude of conformations, and these complex structural motifs may be potential binding pockets for specific drug recognition sites that need to be understood in details.

**Figure 1:**
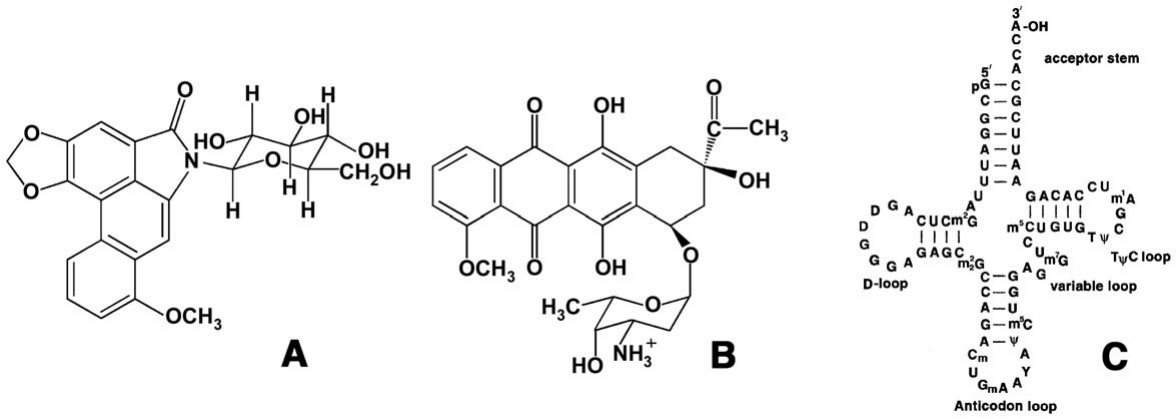
Chemical structures. (A) aristololactam–β-D-glucoside and (B) daunomycin and (C) the cloverleaf structure of yeast tRNA^phe^.

Aristololactam-β-D-glucoside (ADG, Fig. 1A) is an alkaloid of the *Aristolochia* group that have attracted recent attention for their anti tumoral activites [24], [25]. The alkaloid has close resemblance to the clinically used anticancer agent daunomycin (DAU, Fig. 1B) as both have planar structuresl carrying sugar moieties, and bind to double stranded DNA by intercalation exhibiting guanine-cytosine base pair specificity [26]–[28]. The clinical importance and biotarget of ADG is still not established although many studies have revealed its potential biological utility [24], [29]. For such an effective DNA binder with close structural similarity to daunomycin, it is of great importance to obtain detailed information on the binding characteristics to RNA and this prompted us to undertake a comparative interaction study of this alkaloid and DAU with tRNA^Phe^ (tRNA hereafter) (Fig. 1C) from a variety of biophysical studies. tRNA represents one of the most common and most thoroughly characterized natural RNAs. They are versatile molecules with unique cloverleaf structures exhibiting high degree of folding, their structures being stabilized by base pairing and other tertiary interactions. They constitute about 15% of the total cellular RNA and bind to the A-site of ribosomes. Recently there has been an increasing interest in understanding the binding of many small molecules to tRNA [30]–[33].

## Results and Discussion

### Spectrophotometric studies and elucidation of binding affinity

**Figure 2:**
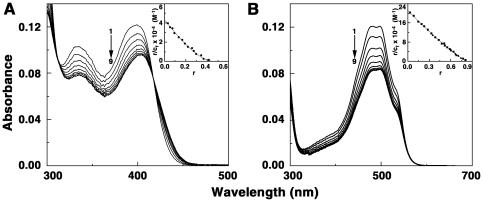
Absorption spectral titration of drugs with tRNA. (A) ADG (11 µM) treated with 0, 55, 110, 165, 220, 330, 440, 550 and 660 µM (curves 1–9) of tRNA and (B) DAU (10.4 µM) treated with 0, 20.8, 52, 83.2, 124.8, 166.4, 208, 312 and 364 µM (curves 1–9) of tRNA. Inset: representative Scatchard plot of each complexation. The solid lines represent the non-linear least square best fit of the experimental points to the neighbour exclusion model.

In the visible absorption spectral region (300–600 nm) both ADG and DAU have characteristic peaks that could be monitored to understand the binding of these molecules to tRNA. The changes in the absorption spectra may be conveniently used to monitor the interaction. In Fig. 2A–B absorption spectral changes in ADG and DAU on titration with increasing concentration of tRNA are presented. The spectrum marked ‘1’ is the absorption spectra of free drug molecules in each case. All the absorption spectral titrations showed hypochromic and bathochromic effects. Titration of tRNA with ADG (Fig. 2A) showed hypochromic change at 398 nm gradually with increasing P/D (nucleotide phosphate/ligand molar ratio) and a slight bathochromic shift of 4–5 nm with an isosbestic point at 417 nm. At saturating P/D, the change in hypochromicity was about 23%. On the other hand, DAU-tRNA showed larger hypochromic effect at 480 nm with increasing P/D, with a red shift of about 16 nm and hypochromicity change of about 31% at saturation (Fig. 2B). The results of the spectrophotometric titration data were expressed as Scatchard plots that were analyzed further by the McGhee-von Hippel methodology [34] for non cooperative binding for evaluation of the binding constants The Scatchard plots are depicted in the inset of Fig. 2. From this analysis, it was found that the binding affinity values (K′) of ADG and DAU to tRNA were 4.33×10^4^ M^−1^ and 2.12×10^5^ M^−1^, respectively. The number of binding sites was around two and one, respectively, for ADG and DAU. These values are presented in Table 1. The binding affinity of DAU to tRNA is higher and of the order of 10^5^ M^−1^ while the binding affinity of ADG was smaller compared to DAU.

**Table 1:** Binding parameters of tRNA complexation with ADG and DAU obtained from spectrophotometric and spectrofluorimetric studies in CP buffer 20 mM [Na^+^] at 20°C.

Drug	Spectrophotometry		Spectrofluorimetry	
	*K*′/10^4^ M^−1^	n	*K*′/10^4^ M^−1^	n
ADG	4.33±0.20	2.13±0.30	6.38±0.20	2.25±0.05
DAU	21.20±0.30	1.20±0.20	18.58±0.32	1.31±0.10

All the data presented are average of four determinations.

### Fluorescence spectral titration and quenching studies

**Figure 3:**
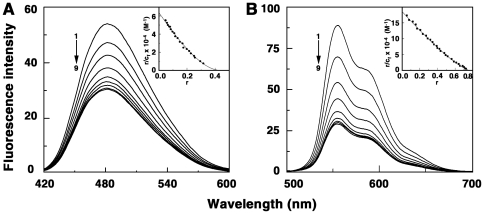
Fluorescence spectral titration of drugs and tRNA. (A) ADG (3.07 µM) treated with 0, 15.35, 30.7, 46.05, 61.4, 92.1, 122.8, 168.85 and 214.9 µM (curves 1–8) of tRNA and (B) DAU (3.0 µM) treated with 0, 15, 30, 45, 60, 75, 90, 105 and 120 µM (curves 1–8) of tRNA.

ADG and DAU are strongly florescent molecules. Their emission spectra are in the 400–600 and 500–700 nms regions, respectively, with maximum around 483 and 556 nm when excited around 400 and 480 nms. Complexation was monitored by titration studies keeping a constant concentration of the drug and increasing the concentration of tRNA. A progressive quenching of the fluorescence was observed for both ADG and DAU, eventually reaching the saturation point without any shift in the wavelength maximum (Fig. 3A–B). But the extent of quenching was significantly different in each case. Maximum fluorescence quenching (67%) was observed in DAU-tRNA system (Fig. 3B) indicating a strong association of the DAU molecules to the tRNA whereas quenching of ADG fluorescence (Fig. 3A) was comparatively lower, at about 43%. Thus, the fluorescence titration studies indicated that a stronger quenching was observed for the DAU system. Analysis of the data through Scatchard and McGhee-von Hippel methodology gave binding affinity values (Table 1) close to that obtained from spectroscopy results. This trend in the binding affinity values was similar to that obtained from absorption spectral studies.

The Stern-Volmer quenching constant (*K*_sv_) of the fluorescence of ADG and DAU by tRNA calculated from the fluorescence spectra revealed values of (1.15±0.12)×10^4^ and (3.90±0.12)×10^4^ M^−1^, respectively. The quenching constants also indicate the strong bimolecular binding process between the drugs and tRNA over other unimolecular photophysical processes. This study underscores the significant affinity of both compounds to tRNA and the higher affinity of DAU over ADG.

**Figure 4:**
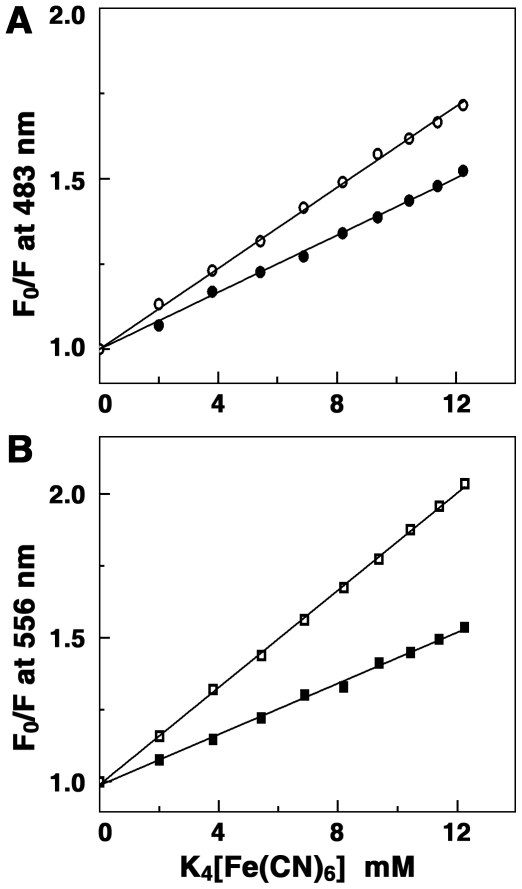
Stern-Volmer plots for the quenching of the fluorescence of drugs-tRNA complex by K_4_[Fe(CN)_6_]. (A) ADG and (B) DAU. Symbols are in absence (○) ADG, (□) DAU and, in presence (•) ADG and (▪) DAU of tRNA.

The fluorescence quenching method is a reliable method for studying the binding mode of small molecules to nucleic acids is. Molecules bound to the surface of the polyanionic macromolecule will be easily accessible to a quencher while those buried inside the helix will be protected from the quencher [35], [36]. An anionic quencher like [Fe(CN)_6_]^4−^ cannot access the inside of the helix due to the electrostatic barrier from the negatively charged phosphate groups and consequently very little quenching will be observed in case of small molecules bound in the interior of the tRNA helix. Consequently, the magnitude of the Stern-Volmer quenching constant (*K*_sv_) of ligands that are bound inside will be lower than that of the free molecules. In Fig. 4, the data on the fluorescence quenching of ADG-tRNA and DAU-tRNA complexes in presence of K_4_[Fe(CN)_6_ is presented. *K*_sv_ values of free ADG and DAU with [Fe(CN)_6_]^4−^ were 59.0 and 84.3 M^−1^, respectively, while that of bound ones were 41.0 and 44.2 M^−1^, respectively, for ADG and DAU indicating that the bound ADG and DAU molecules are considerably protected and sequestered away from the solvent. This result suggests an intercalative or similar kind of binding inside the tRNA helix. The binding of daunomycin to tRNA was previously investigated by Shafer by thermal melting and fluorescence studies and intercalation was suggested [37]. The present data further reveals a more efficient binding of DAU compared to ADG.

### Determination of quantum efficiency

The quantum efficiency (Q) of a nucleic acid binding ligand is a measure of the energy transferred from the nucleic acid to the ligand upon complexation and is evaluated from the ratio of the quantum efficiency of ligand complexed to nucleic acid (q_b_) to the quantum efficiency of the free ligand (q_f_). Determination of quantum efficiency [38] further supports the strong binding of ADG and an even stronger binding of DAU to tRNA. A plot of ΔAbsorbance against the inverse of tRNA concentration gave an exponential plot (not shown) from which quantum efficiency values of 0.68 and 0.46, respectively, have been determined for ADG and DAU. It can be observed that Q is <1.0 and this denotes that both ADG and DAU exhibit greater fluorescence when free in solution compared to the bound state, i.e. the loss of fluorescence energy is more efficient when bound to the tRNA helix. This observation suggests that the loss of fluorescence energy through interactions with the solvent could occur more efficiently from the free ligand than the bound ligand.

### Binding stoichiometry (Job plot)

**Figure 5:**
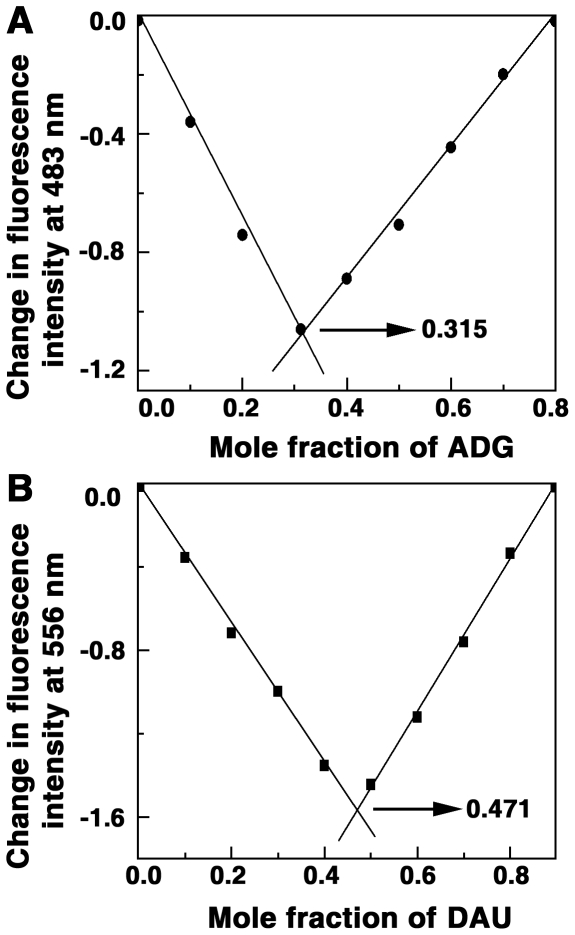
Job's plot for the binding of ADG and DAU with tRNA. (A) ADG and (B) DAU.

The binding stoichiometry of association of ADG and DAU with tRNA and the possible number of binding sites were determined independently by continuous variation analysis (Job plot) [39] in fluorescence. In Job plot, the ligand: RNA molar ratio is varied while the total molar concentration remains constant. The stoichiometry of binding is determined by the molar ratio where maximal binding is observed. The plot of difference fluorescence intensity (ΔF) at 483 and 556 nms, respectively for ADG and DAU, versus their mole fractions presented in Fig. 5, revealed a single binding mode for both these molecules on the tRNA. From the inflection points χ = 0.315 and 0.471 (indicated by arrow), the number of nucleotides bound per ADG and DAU can be estimated to be around 2.17 and 1.12, respectively. This is in good agreement with the number of binding sites obtained from the non-cooperative McGhee-von Hippel analysis of the spectrophotometric data (Table 1).

### Circular dichroic spectral studies

**Figure 6:**
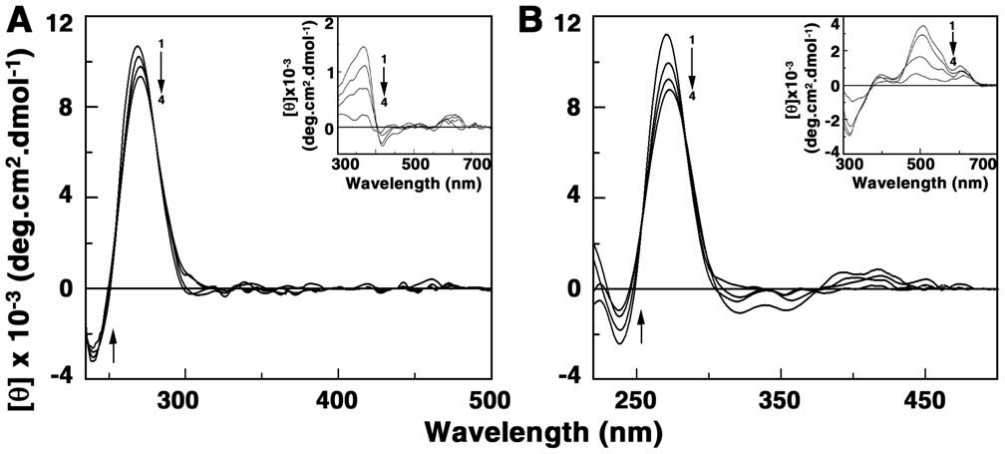
Circular dichroic spectra of tRNA-drug complexes. tRNA (60 µM) treated with (A) 30, 60, 90 and 108 µM (curves 1–4) of ADG and (B) 18, 36, 60 and 90 µM of DAU. Inset: Induced circular dichroic spectra of ADG and DAU, respectively.

The conformational changes on tRNA associated with the binding of ADG and DAU were probed through circular dichroic (CD) studies. The CD spectrum of tRNA was characterized by a large positive band around 270 nm (curve 1, Fig. 6A–B) that is typical of an A-form RNA conformation. There was a moderate decrease in the ellipticity of this positive band as the binding progressed, indicating most likely a disruption of the base stacking interactions, and the extent of change was larger in DAU compared to ADG. To see whether the binding induced any optical activity in the bound drug molecules, a study of induced CD in the 300–600 nm region was carefully performed by keeping a constant concentration of the drugs and varying the concentration of tRNA. Induced CD indicates the CD of the ligand in its absorption region where the RNA does not have any contribution and is generated due to the asymmetric arrangement of the bound molecules on the helix. The result of such study is presented in the insets of Fig. 6A and B. The induced CD spectrum with increasing tRNA, denoted as curve 1 was characterized by a minimum at 310 nm. The ellipticity of this band decreased as the P/D decreased. This study indicated the strong chiral environment of the bound molecules in the stranded organization of tRNA and is due to the effective coupling of the transition moments of the bound drug with the transition moments of the chirally arranged base/base pairs. It may be noted that ADG has no CD in the 200–4000 nm region while DAU has a weak intrinsic CD in the region that enhanced in ellipticity on binding to tRNA.

### Isothermal titration calorimetry

**Figure 7:**
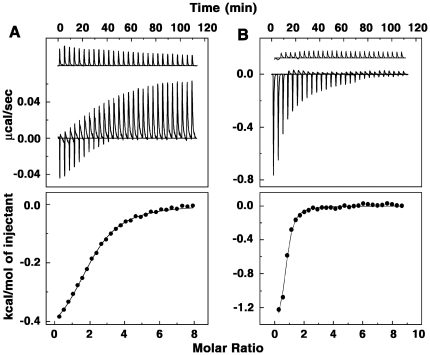
ITC profiles for the binding of (A) ADG and (B) DAU with tRNA. The top panels represent the raw data for sequential injection of tRNA into drug solution and the bottom panels show the integrated heat data after correction of heat of dilution against molar ratio of poly(A)/drug. The data points (closed circle) were fitted to a one site model, and the solid lines represent the best fit data.

Isothermal titration calorimetry (ITC) is an effective tool to thermodynamically characterize the binding of small molecules to macromolecules [40], [41]. ITC essentially provides insight into the energetics of the complexation along with quantitative values of the binding affinity and stoichiometry of the reaction. Because ITC measures heat exchange it provides a tool independent of the spectroscopic changes that occur in the reaction. Therefore, this technique was used to characterize the thermodynamic profiles of tRNA-ADG and tRNA-DAU complex formation. The top panel of Fig. 7 presents the representative primary data from the calorimetric titration of tRNA into solutions of ADG and DAU at 20°C. Each heat burst curve in the figure corresponds to a single injection. These injection heats were corrected by subtracting the corresponding dilution heats derived from the injection of identical amounts of tRNA into the buffer alone (upper panels, curves off set for clarity). In Fig. 7 (bottom panel) the resulting corrected heats are plotted against the molar ratio. The data points in this panel reflect the experimental points and the continuous line represents the calculated best fit to the data. It can be seen that the binding was characterized by exothermic heats. The ITC data were fit to a single site model as the integrated heat data showed only one binding event. The result yielded a binding affinity (*K*) value of (6.50±0.04)×10^4^ M^−1^, a free energy change (Δ*G*) of −6.44 kcal/mol, an enthalpy change (Δ*H*) of −1.02 kcal/mol, an entropy contribution (*T*Δ*S*) of 5.42 kcal/mol and a binding site size of 2.10 nucleotides for ADG and a *K* of (2.22±0.05)×10^5^ M^−1^, ΔG of −7.14 kcal/mol, Δ*H* of −1.42 kcal/mol and entropy of −5.72 kcal/mol for DAU (Table 2). The overall binding affinities observed from ITC and the *K′* values from spectroscopic binding are comparable and the trend is the same. Energetics of the interaction indicates that the binding was favored by small negative enthalpy and large positive entropy changes in both the cases. The large positive entropy term is suggestive of the disruption and release of water molecules on intercalation of ADG and DAU with sugar moieties into the RNA helix. There are many examples in the literature for entropy driving nonspecific small molecule–DNA and RNA interactions that essentially involve the release of solvent and counterions from the interface [42], [43]. Specific examples are actinomycin and netropsin interaction with DNA, quinacrine and sanguinarine interactions with poly(A) etc. [42], [43]. However, it may be noted that previous reports on the thermodynamic profile of daunomycin-DNA interaction suggested an enthalpically driven binding and not entropy favoured one [42], . It s likely that the structural difference between tRNA and DNA may be responsible for this difference in the energetics of the interaction.

**Table 2:** ITC derived thermodynamic profiles for the binding of ADG and DAU to tRNA at different Na+ concentrations in CP buffer at 20°C.

Drug	NaCl (mM)	*K/*10^4^ M^−1^	N	Δ*G/*kcal mol^−1^	Δ*H/*kcal mol^−1^	*T*Δ*S/*kcal mol^−1^	Δ*G_t_/*kcal mol^−1^	Δ*G_pe_/*kcal mol^−1^	ZΨ
	10	9.9±0.5	2.04±0.02	−6.74±0.05	−1.35±0.01	5.40±0.01	5.31±0.01	1.43±0.02	
ADG	20	6.5±0.3	2.10±0.02	−6.50±0.04	−1.02±0.01	5.42±0.01	5.28±0.02	1.21±0.03	−0.53±0.04
	50	4.2±0.1	2.40±0.03	−6.24±0.02	−0.58±0.02	5.62±0.02	5.31±0.02	0.93±0.03	
	10	38.5±0.3	1.09±0.02	−7.54±0.02	−1.71±0.02	5.71±0.01	5.03±0.01	2.51±0.02	
DAU	20	22.2±0.1	1.15±0.05	−7.21±0.03	−1.42±0.02	5.72±0.02	5.08±0.02	2.13±0.01	−0.93±0.01
	50	8.40±0.2	1.36±0.02	−6.64±0.03	−0.48±0.02	6.12±0.03	5.01±0.01	1.63±0.01	

The data in this table are derived from ITC experiments conducted in CP buffer. *K* and Δ*H* values were determined from fits of the ITC profiles, with indicated errors reflecting standard deviations of the experimental data from the fitted curves. The value of Δ*G* were determined using the equation ΔG = −(*RT*ln*K*). The values of *T*Δ*S* were determined using equation *T*Δ*S* = Δ*H*-Δ*G*. The uncertainties in the determinations are indicated. The ITC profiles were fit with a model for single binding site.

### Dependence of binding on the ionic strength

Previously published data from our laboratory suggested that electrostatic interactions are not so important for tRNA binding of isoquinoline alkaloids [20]. On the contrary, the binding of aminoglycosides to RNA have been reported to be driven by strong electrostatic interactions [46], [47]. We studied the effect of salt concentration on the binding of ADG and DAU to tRNA in conjunction with van't Hoff analysis through calorimetric experiments. As the [Na^+^] concentration was increased from 10 mM to 50 mM [Na^+^], the binding affinity of the interaction decreased from 9.9×10^4^ M^−1^ to 4.2×10^4^ for ADG and from 3.85×10^5^ M^−1^ to 8.4×10^4^ M^−1^ for DAU (Table 2). Thus the binding of both ADG and DAU are thermodynamically linked to Na+ concentration. Polyelectrolytic theories based on Manning's counter ions condensation model describe the process and provide a basis for interpreting this data [48]. From polyelectrolytic theory, the slope of the best fit line for a plot of log *K* versus log [Na+] is related to the counterion release [42]

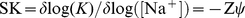
(1)where SK is equivalent to the number of couterions released upon binding of a drug, Z is the apparent charge of the bound ligand per phosphate binding and ψ is the fraction of [Na^+^] bound per phosphate group. The slope of the plot of log *K* versus log [Na^+^] (Fig. S1) gave values of −0.53 and −0.93, respectively, for ADG and DAU. The values of Zψ reported for daunomycin-DNA complexes are in the range 0.84–1.08 [44], [49]. These values represent the counter ions released per phosphate upon binding and suggest stronger electrostatic contact of DAU over ADG with tRNA. This has been further verified by partitioning the electrostatic (Δ*G*_pe_) and nonelectrostatic (ΔG_t_) contributions to the Gibbs energy (Fig. S2). The polyelectrolytic contribution to the overall observed free energy can be quantitatively estimated from the relationship
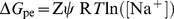
(2)at a given salt concentration and the non electrostatic contribution can be calculated as the difference between ΔG and Δ*G*_pe_. Here Zψ is the slope of the van't Hoff plot. At 50 mM [Na^+^], the contribution of Δ*G*_pe_ have been determined to be 1.63 kcal/mol and 0.93 kcal/mol, respectively, for DAU and ADG. These are about 25 and 15%, respectively, of the total free energy change. The positively charged DAU exhibits high level of electrostatic contribution compared to ADG. In case of ADG, it has been suggested that a weak transient positive charge may be developed at the N6 position in presence of nucleic acids [50], [51]. It is likely that such transient charge development is the cause of the observed weak but significant electrostatic contribution to the binding. Clearly the electrostatic contributions to the binding of both DAU and ADG to tRNA are much lower than the contributions from the non electrostatic forces.

### Heat capacity change of binding

**Figure 8:**
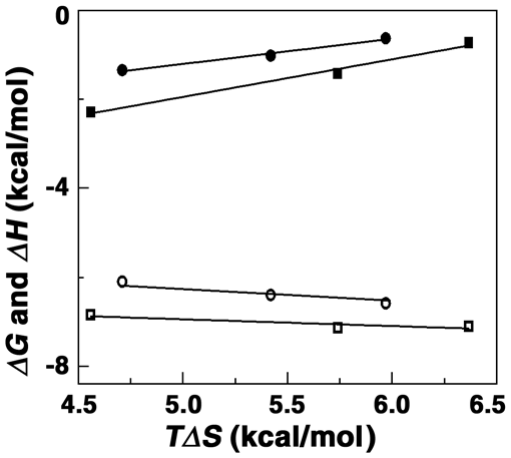
Plot of variation of thermodynamic parameters. *ΔG* (○, □) and *ΔH* (•, ▪) verses *TΔ*S for the binding of ADG and DAU to tRNA.

Temperature dependence of the binding enthalpy measurement allows the determination of the change in heat capacity (Δ*C_p_*) using the equation Δ*C_p_* = δ(Δ*H*)/δ*T*. Heat capacity provides a link between the structural and energetic data and may describe hydration-dehydration effects that occur during the binding process. Temperature dependence ITC was performed in the range 10 to 25°C and the thermodynamic parameters are elucidated at each temperature. These data are presented in Table 3. The association constant varied from (1.18±0.08)×10^5^ M^−1^ at 10°C to (2.90±0.2)×10^4^ M^−1^ at 25°C for ADG and from (3.00±0.07)×10^5^ at 10°C to (1.26±0.03)×10^5^ M^−1^ at 25°C for DAU. Essentially, the binding affinity decreased by about four times over the temperature range for ADG and about three times for DAU. The binding enthalpy values increased and the favorable entropy contribution decreased with increasing temperature. Nevertheless, only small changes occurred in the free energy values. The reaction enthalpy and entropy, both of which were strong functions of temperature, compensate to make the reaction free energy more or less independent of temperature (Fig. 8). Such compensation was observed for many biomolecular interactions [52]–[54] and suggests the involvement of significant hydrophobic interactions in the binding. The temperature dependence of the enthalpy change was used to estimate change in heat capacity (Δ*C_p_*). The slope of the line revealed a value of −47 and −99, respectively, for ADG and DAU (Table 3). Negative heat capacity values have been observed for a variety of small molecules binding to DNA [42], [54]–[58] and RNA [19], [52]. Change in solvent accessible surface area (SASA) has been shown to be a major component of Δ*C_p_* [59]–[61]. Further, structured water like the water of hydrophobic hydration can be associated with large heat capacity and the release of such water on transfer of the non-polar groups into the interior of the helix can contribute a negative term to the Δ*C_p_*. Murphy and Churchill have discussed four types or modes of DNA recognition by small molecules, which are sequence specific, nonspecific, minimal sequence specific and structure specific [55]. Small negative Δ*C_p_* values have been implicated to be associated with a minimal sequence specific binding reactions. The slightly negative non-zero Δ*C_p_* value that is observed for ADG and DAU-tRNA complexation appears to denote structure specific binding. It is known that for DNA intercalators and groove binders a large hydrophobic contribution to the binding free energy is expected from their aromatic ring system and binding should be energetically favorable [62]. From the Records relationship, Δ*G*_hyd_ = 80(±10)×Δ*C_p_*, the free energy contribution from the hydrophobic transfer step of binding of these molecules may be calculated [62]. The Δ*G*_hyd_ values for ADG and DAU binding to tRNA were deduced to be −3.8 and −7.9 kcal/mol, respectively, well within the range observed for intercalating molecules [58], [59].

**Table 3:** ITC derived thermodynamic profiles for the binding of ADG and DAU to tRNA at different temperatures in CP buffer 20 mM [Na^+^].

Drug	T(K)	*K*/10^5^ M^−1^	N	Δ*G*kcal mol^−1^	Δ*H*kcal mol^−1^	*T*Δ*S*kcal mol^−1^	Δ*C_p_/*cal mol K
	283	1.18±0.01	2.05±0.01	−6.60±0.01	−0.63±0.01	5.97±0.02	
ADG	293	0.65±0.03	2.10±0.02	−6.44±0.03	−1.02±0.01	5.42±0.02	−47±2
	298	0.29±0.02	2.54±0.05	−6.06±0.03	−1.35±0.02	4.71±0.03	
	283	3.00±0.01	1.08±0.01	−7.10±0.01	−0.73±0.01	6.37±0.02	
DAU	293	2.22±0.01	1.15±0.01	−7.14±0.02	−1.42±0.02	5.72±0.01	−99±3
	298	1.26±0.02	1.21±0.02	−6.85±0.04	−2.29±0.05	4.56±0.02	

All the data in this table are derived from ITC experiments. *K* and *ΔH* values were determined from fits of the ITC profiles, with indicated errors reflecting standard deviations of the experimental data from the fitted curves. The value of Δ*G* were determined using the equation Δ*G* = −(*RT*ln*K*). The values of *T*ΔS were determined using equation *T*Δ*S* = Δ*H*-ΔG. The uncertainties in the determinations are indicated. The ITC profiles were fit with a model for single binding site.

### Thermal melting studies

**Figure 9:**
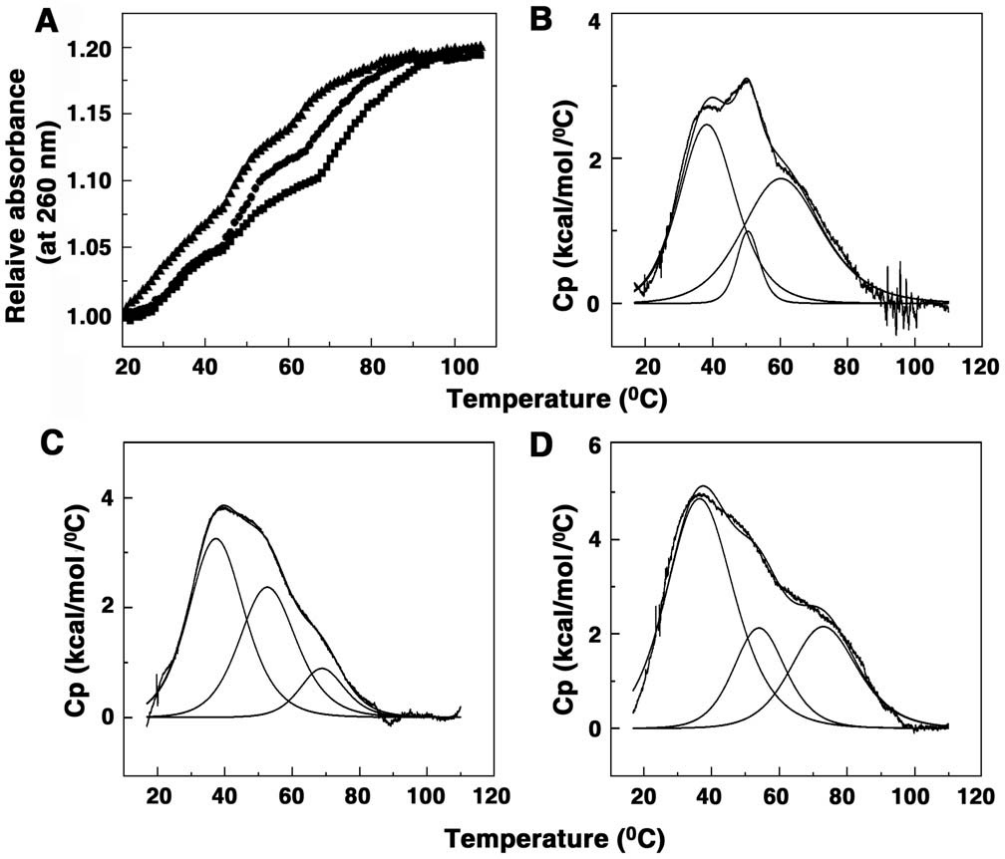
Optical and DSC melting profiles of tRNA-drug complexes. (A) optical melting of tRNA alone (▴), in presence of ADG (•) and in presence of DAU (▪), (B) DSC thermograms of tRNA, (C) tRNA-ADG complex and (D) tRNA-DAU complex.

The ability of ADG and DAU to enhance the stability of tRNA on complexation was investigated from optical melting and differential scanning calorimetry studies. The native tRNA exhibits an optical melting profile (Fig. 9A) with three transitions that has been stabilized by the presence of bound molecules. The DSC thermograms of tRNA can be deconvoluted to three transitions with *T*_m_ values of 38, 50 and 61°C, respectively (Fig. 9B). These are in agreement with the previous reports [20], [63], [64]. The transitions were reversible but the non unity value for the ratio of the calorimetric (Δ*H*_cal_) and van't Hoff (Δ*H*_van't Hoff_) enthalpy indicated that the melting of the tRNA may not be obeying a simple two state transition process. The melting of transition I was not affected on binding of ADG and DAU. ADG stabilizes the second transition by about 2°C while a higher stabilization of about 4°C was effected by DAU. A larger stabilization of 7°C by ADG and 12.5°C by DAU was effected on the third transition (Fig. 9 C,D). This result further reiterates the strong binding of DAU to tRNA compared to ADG.

### Conclusions

Interaction of the sugar containing molecules aristololactam-β-D-glucoside and daunomycin with tRNA^phe^ was investigated. Scatchard plots from absorption and fluorescence titrations revealed that both the compounds bind to tRNA non cooperatively. Fluorescence quenching studies suggested both ADG and DAU are bound inside the helical organization of the tRNA, most likely by intercalation, but the former is bound more tightly. A large favorable component for electrostatic contribution to the binding of ADG and DAU to tRNA was revealed from the salt dependent data and the dissection of the free energy term that correlated with the entropic contribution. The binding of ADG and DAU to tRNA was predominantly entropy driven with a smaller favorable enthalpy term that enhanced with temperature. The small but negative heat capacity change of −47 and −99 cal/mol K and the significant enthalpy-entropy compensation phenomenon observed confirmed the involvement of multiple weak noncovalent interactions in the binding process. Both the drugs enhanced the thermal stability of tRNA. Circular dichroism studies provided evidence for a small but significant perturbation of the tRNA structure. Taken together, the results suggest that the binding of these molecules on the tRNA^phe^ structure appears to be mostly by partial intercalation, but with significant contribution from electrostatic forces. Many well-known molecules like aminoglycosides bind RNA with strong electrostatic interactions [46], [47]. Since molecular recognition of RNA by small molecules is an area of immense current interest, our present results may contribute to the development of better small molecule based RNA therapeutic agents.

## Materials and Methods

### Biochemicals

tRNA^Phe^ (yeast) and daunomycin were purchased from Sigma-Aldrich Corporation (St. Louis, MO, USA). The ratio of the absorbance at 260 to 280 nm was around 1.85 for tRNA indicating that the sample was free from protein contaminations. Aristololactam- β-D-glucoside was extracted in our laboratory from *Aristolochia indica* and crystallized from ethanol [65], [66].

### Preparation of stock solutions

tRNA solution was prepared in the experimental buffer and dialyzed in cold room. Its concentration was determined spectrophotometrically using a molar extinction coefficient (ε) of 6900 (M^−1^ cm^−1^) at 260 nm expressed in terms of nucleotide phosphates [20], [64]. Stock solutions of ADG and DAU were prepared in the experimental buffer and kept protected in the dark till use. The concentrations of ADG and DAU were determined by absorbance measurement using molar extinction coefficients as follows: ADG-10,930 M^−1^ cm^−1^ at 398 nm (in dimethyl sulfoxide) and DAU-11,500 M^−1^ cm^−1^ at 480 (in aqueous buffer), respectively [50], [51]. No deviation from Beer's law occurred in the concentration range employed in this study. All experiments were conducted in Citrate-Phosphate (CP) buffer (10 mM [Na^+^]), pH 7.0, containing 0.5 mM Na_2_HPO_4_. The pH was adjusted using citric acid. For ADG binding studies the buffer contained additionally 240 mM of DMSO [50]. Glass distilled deionized water and analytical grade reagents were used throughout. pH measurements were made on a Sartorius high precision pH meter with a semi micro electrode (Sartorius AG, Germany) with an accuracy of >±0.01 units. All buffer solutions were filtered through 0.45 µm Millipore filters (Millipore, India Pvt. Ltd, Bangalore, India) before use.

### Absorbance and fluorescence spectroscopy

Electronic absorption spectral titrations were performed on a Shimadzu Pharmaspec 1700 spectrophotometer (Shimadzu Corporation, Tokyo, Japan) at 20±0.5°C using the methodology of Chaires *et al* [67] and described previously in details by us [20], [68]. Briefly, a known concentration of the RNA solution was kept in the sample and reference cuvettes and small aliquots of a known concentration of the drug was titrated into the sample cuvette. After each addition, the solution was thoroughly mixed and allowed to reequilibrate before noting the absorbance at the wavelength maximum and the isosbestic point. The data obtained from these titrations were utilized for constructing Scatchard plots. Steady state fluorescence measurements were performed on a Hitachi F4010 fluorescence spectrometer (Hitachi Ltd., Tokyo, Japan) or Shimadzu RFPC 5301 spectrofluorimeter in fluorescence free quartz cuvettes of 1 cm path length as described previously [69]. The excitation wavelengths for ADG and DAU were 350 nm and 424 nm, respectively. All measurements were performed keeping excitation and emission band passes of 5 nm. The sample temperature was maintained at 20±1.0°C using Eyela Uni Cool U55 water bath (Tokyo Rikakikai Co. Ltd., Japan).

### Binding affinity calculations

The amounts of free and RNA bound drug were determined as follows. In absorbance, after each addition of the drug to the tRNA solution (40 µM), the absorbance data at the respective isosbestic points were noted and the total drug concentration present was calculated as C_t_ = A_iso_/ε_iso_. This data was then used to calculate the expected absorbance at wavelength maximum as A_exp_ = C_t_ε_λmax_. The difference in A_exp_ and the observed absorbance (A_obsd_) was then used to calculate the amount of bound drug as C_b_ = ΔA/Δε = (A_exp_−A_obsd_)/(ε_f_−ε_b_). The free drug concentration was determined by difference, C_f_ = C_t_−C_b_. The extinction coefficient of the completely bound drug was determined by adding a known quantity of the drug to a large excess of tRNA and on the assumption of total binding, ε_b_ = A_max_/C_t_. Alternatively, the absorbance of a known quantity of drug was monitored at the wavelength maximum while adding known amounts of RNA until no further change in absorbance was observed. Both these protocols gave values within experimental errors. In fluorescence, the amount of bound drug was calculated from the relation C_b_ = C_t_(I−I_o_)/(V_o_−1)I_o_, where C_t_ is the known total drug concentration, I is the observed fluorescence, I_o_ is the fluorescence intensity of identical concentration of drug in absence of RNA and V_o_ is the experimentally determined ratio of the fluorescence intensity of totally bound drug to that of free drug. Free drug concentrations (C_f_) were obtained from the relationship C_t_ = C_b_+C_f_. The binding ratio (r) is defined as r = C_b_/[RNA]_total_. Binding data obtained from spectrophotometric and spectrofluorimetric titrations were converted to Scatchard plots of r/C_f_ *versus* r. The Scatchard plots showed negative slopes at low r values as observed for noncooperative binding isotherms. Hence the plots were analyzed using the noncooperative McGhee-von Hippel equation [34]

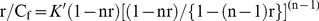
(1)where *K′* is the intrinsic binding constant to an isolated binding site and n is number of nucleotides excluded by the binding of a single drug molecule. The binding data were analyzed using the Origin 7.0 software (Microcal, Inc., Northampton, MA, USA) that determines the best-fit parameters to equation (1).

### Binding stoichiometry determination

Job's continuous variation method [39] was employed to determine the binding stoichiometry in each case using fluorescence spectroscopy. At constant temperature, the fluorescence signal was recorded for solutions where the concentrations of both RNA and drugs were varied while the sum of their concentrations was kept constant. The difference in fluorescence intensity (ΔF) of the drugs in the absence and presence of RNA was plotted as a function of the input mole fraction of each drug as reported previously [43]. The break point in the resulting plot corresponds to the mole fraction of the bound drug in the complex. The stoichiometry was obtained in terms of RNA-alkaloid [(1-χ_alkaloid_)/χ_alkaloid_] where χ_alkaloid_ denotes the mole fraction of the respective alkaloid. The results reported are averages of at least three experiments.

### Fluorescence quenching and quantum efficiency measurements

Fluorescence quenching studies were carried out with the anionic quencher [Fe(CN)_6_J^4−^. The quenching experiments were performed by mixing, in different ratios, two solutions, one containing KCl and the other K_4_[Fe(CN)_6_], in addition to the normal buffer components at fixed total ionic strength. Fluorescence quenching experiments were performed at a constant P/D (RNA base nucleotide/alkaloid molar ratio), monitoring fluorescence intensity changes as a function of changing concentration of ferrocyanide as described previously [19]. At least four measurements were taken for each set and averaged out. The data were plotted as Stern-Volmer plots of I_o_/I versus [Fe(CN)_6_]^4−^.

Fluorescence quantum efficiency was calculated using the following equation [35]

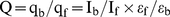
(2)where ε_f_ and ε_b_ represent the molar extinction coefficients of the free and bound drug and I_b_ and I_f_ are the fluorescence intensities of bound and free drug respectively. The quantum efficiency was determined according to the procedure described earlier [19].

### Circular dichroism spectroscopy

Circular dichroism (CD) data were taken on a Jasco J815 spectropolarimeter (Japan Spectroscopic Ltd., Japan) at 20±0.5°C as reported earlier [54], [68]–[69]. Temperature control of the spectral measurements was achieved by a Jasco temperature controller (model PFD425 L/15). A rectangular quartz cuvette of 1 cm path length was used. Each spectrum was averaged from five successive accumulations at a scan rate of 50 nm/min. keeping a bandwidth of 1.0 nm at a sensitivity of 100 milli degree, and was base line corrected and smoothed within permissible limits using the inbuilt software of the unit. The molar ellipticity values [θ] are expressed in terms of either per RNA base nucleotide (210–400 nm) or per bound alkaloid (300–500 nm). The CD unit was routinely calibrated using aqueous solutions of d-10 ammonium camphor sulphonate.

### Isothermal titration calorimetry

All isothermal titration calorimetry experiments were performed using a MicroCal VP-ITC unit (MicroCal Inc., Northampton, MA, USA) using protocols reported in our previous papers [18]–[22], [45]. Aliquots of degassed tRNA solution were injected from the rotating syringe (290 rpm) into the isothermal sample chamber containing each of the drug solution (1.4235 mL). Corresponding control experiments to determine the heat of dilution of tRNA were performed by injecting identical volumes of same concentration of the tRNA into buffer. The duration of each injection was 10 seconds and the delay time between the injections was 240 seconds. The initial delay before the first injection was 60 seconds. Each injection generated a heat burst curve (microcalories per second versus time). The area under each heat burst curve was determined by integration using the Origin 7.0 software (MicroCal) to give the measure of the heat associated with the injection. The heat associated with each tRNA-buffer mixing was subtracted from the corresponding heat associated with tRNA injection to the drug to give the heat of drug binding to the tRNA. The heat of dilution for injecting the buffer into each of the drug alone was observed to be negligible. The resulting corrected injection heats were plotted as a function of the P/D molar ratio, and fit with a model for one set of binding sites, and analyzed using Origin 7.0 software to estimate the binding affinity (*K*), the binding stoichiometry (N), and the enthalpy of binding (D*H*). The free energies (D*G*) were calculated using the standard relationship
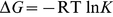
(3)where R is the gas constant (1.987 cal/K mol) and T is the temperature in Kelvin (293 K). The binding free energy coupled with the binding enthalpies derived from the ITC data allowed the calculation of the entropic contribution to the binding (*T*D*S*), where DS is the calculated binding entropy using the standard relationship
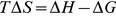
(4)


### Optical melting and differential scanning calorimetry

Absorbance versus temperature profiles (melting curves) of RNA and RNA-alkaloid complexes were measured on the Shimadzu Pharmaspec 1700 unit equipped with the peltier controlled TMSPC-8 model accessory (Shimadzu Corporation, Tokyo, Japan) as described previously [20], [64], [70]. In a typical experiment, the RNA sample (50 µM) was mixed with varying concentrations of the drug under study in the desired degassed buffer in to the eight cell micro optical cuvettes of 1 cm path length. The temperature of the microcell accessory was raised at a heating rate of 0.5°C/min. while continuously monitoring the absorbance change at 260 nm. Melting curves allowed an estimation of melting temperature *T*_m_, the midpoint temperature of the drug bound RNA unfolding process.

To investigate the helix-coil transition, excess heat capacities were measured as a function of temperature on a Microcal VP-differential scanning calorimeter (DSC) (MicroCal Inc., Northampton, MA, USA) as described previously [22]. In a series of DSC scans, both the cells were loaded with the buffer solution, equilibrated at 20°C for 15 minutes, and scanned from 20° to 100°C at a scan rate of 50°C/hour. The buffer scans were repeated till reproducible. On cooling, the sample cell was rinsed and loaded with RNA solution and then with RNA-drug complexes of different molar ratio. Scanning was done in the range 20°–100°C. Each experiment was repeated twice with separate fillings. The DSC thermograms of excess heat capacity versus temperature were analyzed using the Origin 7.0 software. The area under the experimental heat capacity (C_p_) curve was used to determine the calorimetric transition enthalpy (Δ*H*_cal_) given by the equation
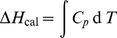
(5)where T is the absolute temperature in Kelvin. This calorimetrically determined enthalpy is model-independent and is thus unrelated to the nature of the transition. The temperature at which excess heat capacity is at a maximum defines the transition temperature (*T*_m_). The model-dependent van't Hoff enthalpy (Δ*H*_v_) was obtained by shape analysis of the calorimetric data and the cooperativity factor was obtained from the ratio (Δ*H*_cal_/Δ*H*_v_). To check the reversibility of the transition, the sample was allowed to cool slowly to 10°C (10°C/h) and a repeat DSC scan was performed on the renatured sample under identical conditions.

## Supporting Information

Figure S1 **The slope of the plot of log** ***K*** **versus log [Na^+^] on the binding of ADG (-•-) and DAU (-▪-) to tRNA.**
(TIF)

Figure S2 **Partitioned polyelectrolytic (Δ** ***G*** **_pe_) (shaded) and nonpolyelectrolytic (ΔG_t_, black) contributions to the Gibbs energy of the complexation of ADG (A) and DAU (B) at different [Na^+^] concentrations.**
(TIF)
