# Supplementary figures and images for: Targeting RNA by Small Molecules: Comparative Structural and Thermodynamic Aspects of Aristololactam-β-D-glucoside and Daunomycin Binding to tRNAphe
Source: PLoS One. 2011 Aug 16;6(8):e23186. doi: 10.1371/journal.pone.0023186 (PMC3156712; doi:10.1371/journal.pone.0023186)

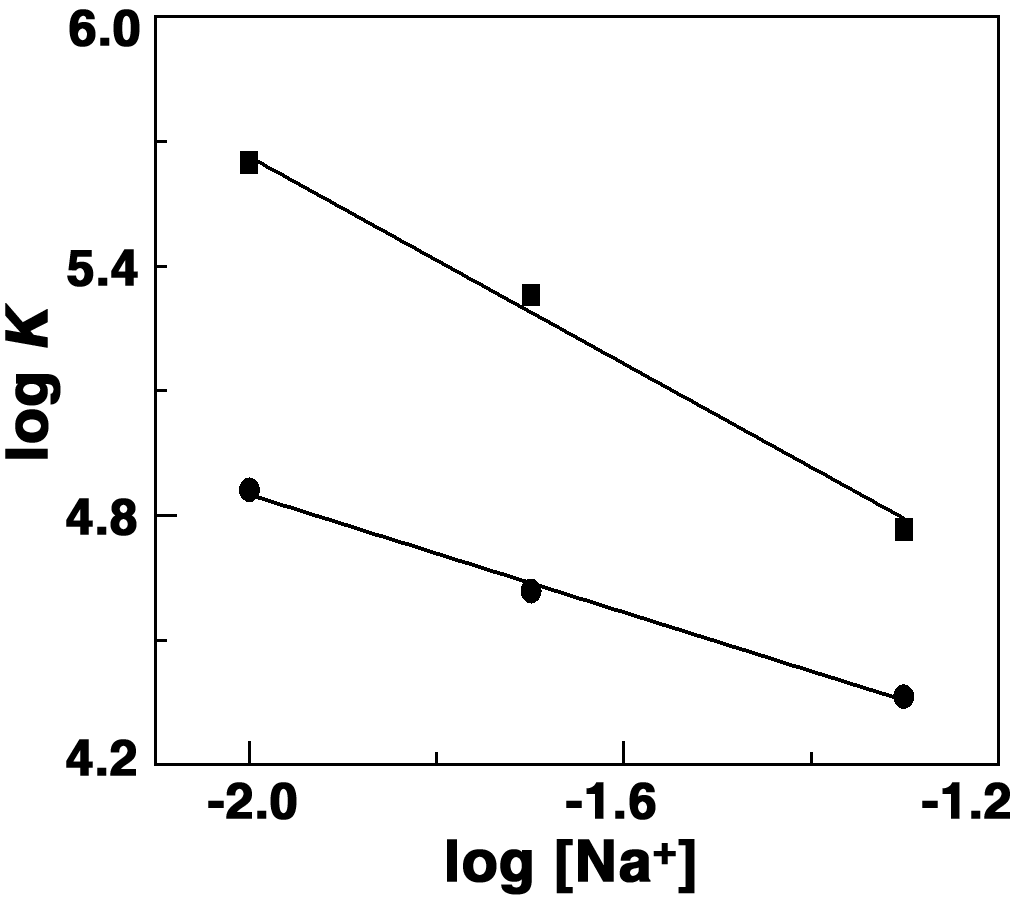

Supplement: Figure S1 — The slope of the plot of log K versus log [Na+] on the binding of ADG (-•-) and DAU (-▪-) to tRNA. (TIF) [file pone.0023186.s001.tif]

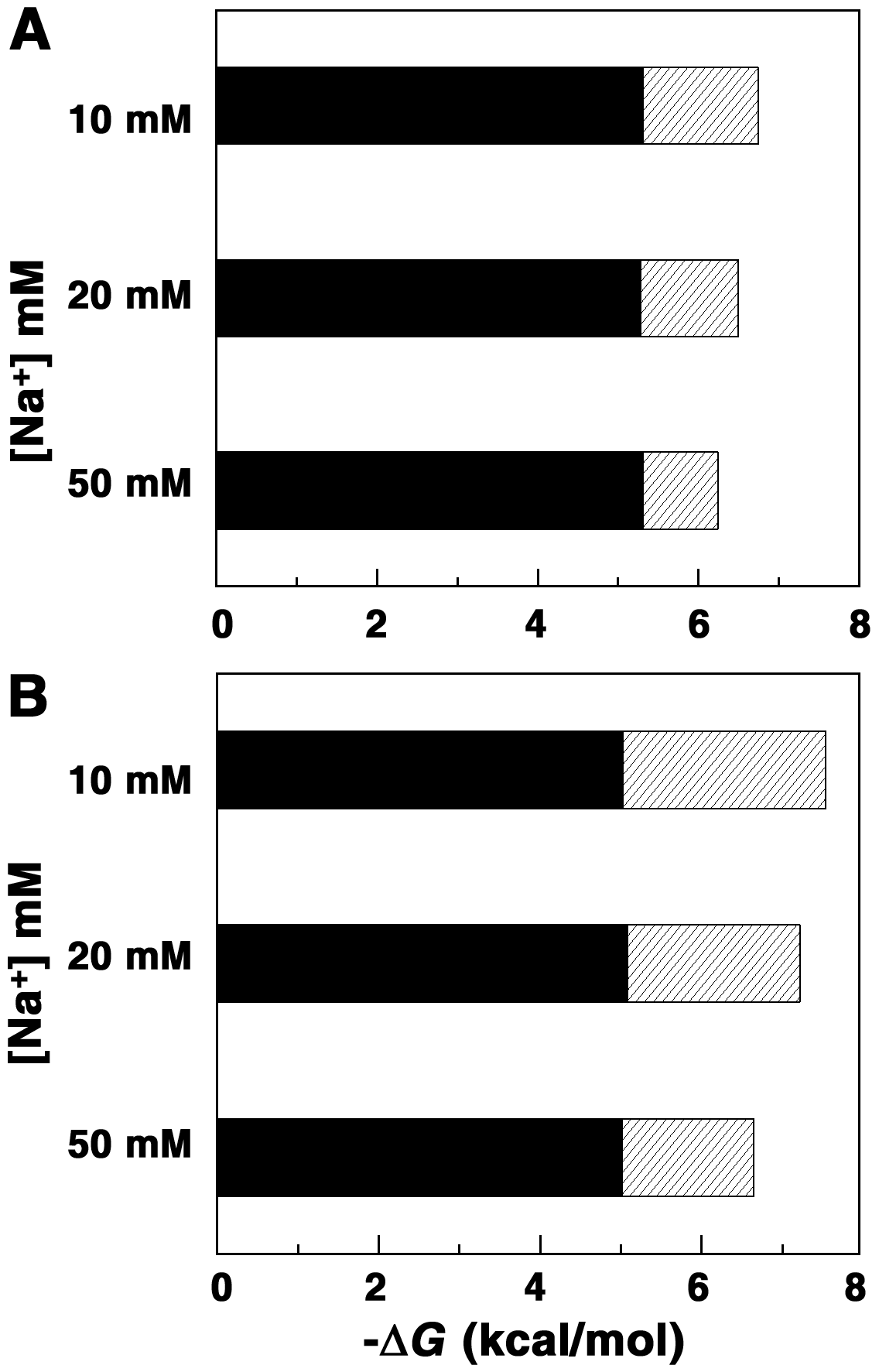

Supplement: Figure S2 — Partitioned polyelectrolytic (Δ G pe) (shaded) and nonpolyelectrolytic (ΔGt, black) contributions to the Gibbs energy of the complexation of ADG (A) and DAU (B) at different [Na+] concentrations. (TIF) [file pone.0023186.s002.tif]
